# Target enzyme mutations are the molecular basis for resistance towards pharmacological inhibition of nicotinamide phosphoribosyltransferase

**DOI:** 10.1186/1471-2407-10-677

**Published:** 2010-12-12

**Authors:** Uffe H Olesen, Jakob G Petersen, Antje Garten, Wieland Kiess, Jun Yoshino, Shin-Ichiro Imai, Mette K Christensen, Peter Fristrup, Annemette V Thougaard, Fredrik Björkling, Peter B Jensen, Søren J Nielsen, Maxwell Sehested

**Affiliations:** 1 Experimental Pathology Unit, Rigshospitalet, Copenhagen, Denmark; 2 TopoTarget A/S, Copenhagen, Denmark; 3 Research Laboratory, Hospital for Children and Adolescents, University of Leipzig, Leipzig, Germany; 4 Department of Developmental Biology, Washington University School of Medicine, St. Louis, MO, USA; 5 Department of Chemistry, Technical University of Denmark, Lyngby, Denmark; 6 Department of Medicinal Chemistry, Faculty of Pharmaceutical Sciences, University of Copenhagen, Copenhagen, Denmark

## Abstract

**Background:**

Inhibitors of nicotinamide phosphoribosyltransferase (NAMPT) are promising cancer drugs currently in clinical trials in oncology, including APO866, CHS-828 and the CHS-828 prodrug EB1627/GMX1777, but cancer cell resistance to these drugs has not been studied in detail.

**Methods:**

Here, we introduce an analogue of CHS-828 called TP201565 with increased potency in cellular assays. Further, we describe and characterize a panel of cell lines with acquired stable resistance towards several NAMPT inhibitors of 18 to 20,000 fold compared to their parental cell lines.

**Results:**

We find that 4 out of 5 of the resistant sublines display mutations of NAMPT located in the vicinity of the active site or in the dimer interface of NAMPT. Furthermore, we show that these mutations are responsible for the resistance observed. All the resistant cell lines formed xenograft tumours *in vivo*. Also, we confirm CHS-828 and TP201565 as competitive inhibitors of NAMPT through docking studies and by NAMPT precipitation from cellular lysate by an analogue of TP201565 linked to sepharose. The NAMPT precipitation could be inhibited by addition of APO866.

**Conclusion:**

We found that CHS-828 and TP201565 are competitive inhibitors of NAMPT and that acquired resistance towards NAMPT inhibitors can be expected primarily to be caused by mutations in NAMPT.

## Background

Drug resistance is a serious concern in the treatment of cancer [1]. It can occur as either *de novo *or acquired resistance following therapy. Besides multi-drug resistance (MDR) caused by ABC efflux pumps, several targeted therapies have described the development of target-specific drug resistance. Thus, up to 90% of the cases of acquired resistance to tyrosine kinase inhibitors are due to over-expression of, or mutations in, the target kinase [2-4]. Acquired resistance can be studied by inducing resistance *in vitro *by growing cells in the presence of increasing concentrations of drug [1].

**Figure 1:**
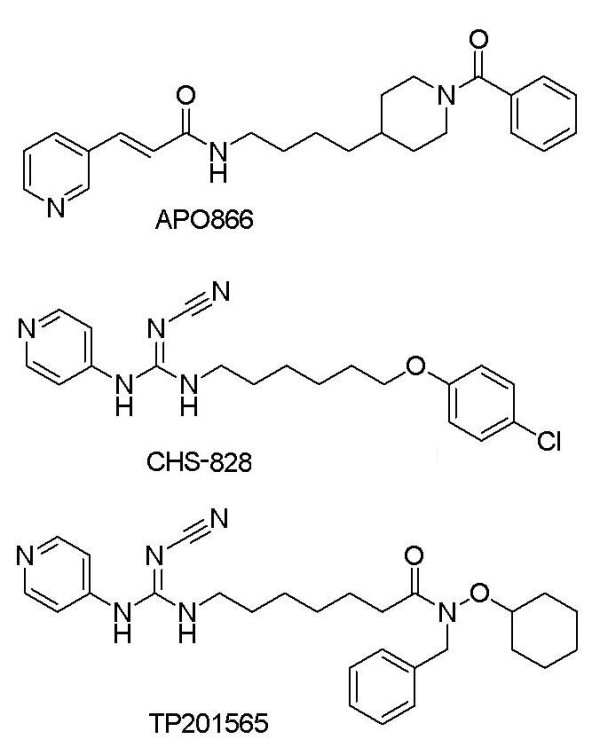
**Chemical structures of APO866, CHS-828 and TP201565**. APO866 and CHS-828 are chemically distinct whereas TP201565 is an analogue of CHS-828.

NAD is an essential cofactor in cell energy production and metabolism as well as the substrate for mono-ADP-ribosyltransferases [5], poly-(ADP-ribose) polymerases (PARPs) [6] and sirtuins [7], all of these converting NAD to nicotinamide. PARPs are involved in DNA repair whereas sirtuins can increase cancer cell survival. To survive under stress and supply metabolites for cell growth malignant cells depend heavily on aerobic glycolysis for generation of ATP [8]. Glycolysis requires relatively more NAD to generate ATP compared to the oxidative phosphorylation normally occurring in non-malignant tissues. Also, cancer cells may display increased expression or activity of PARPs [9-11] and sirtuins [7] for increased DNA repair and cell survival. The first, rate-limiting step in the resynthesis pathway of NAD from nicotinamide is catalyzed by nicotinamide phosphoribosyltransferase (NAMPT) [12]. Nicotinamide is converted to nicotinamide mononucleotide (NMN) using 5-phosphoribosyl-1-pyrophosphate and ATP as substrates. NMN is then converted to NAD by NMN adenyltransferase (NMNAT) [13]. The crystal structure of NAMPT has been resolved and it has been identified as a dimer belonging to the family of type II phosphoribosyltransferases [14-16] - each monomer containing two domains. The dimer contains two binding sites for nicotinamide located in the vicinity of the dimer interface and residues of both monomers may be part of the binding site. Inhibition of NAMPT leads to depletion of NAD [17], secondarily leading to reduction of ATP and later, cell death. Also, it leads to substrate depletion of PARPs and sirtuins and furthermore, both PARPs and sirtuins are inhibited by nicotinamide [18-20]. Tumour cells are more sensitive to NAMPT inhibition and NAD depletion due to increased ATP and NAD consumption [17]. NAMPT inhibition shows high efficacy in haematological malignancies in preclinical studies [21]. APO866 is a specific, competitive, potent inhibitor of NAMPT that displays cytotoxicity in a broad panel of cell lines (Figure 1) [17,22]. APO866 has completed a phase I trial in oncology [22] and is currently undergoing several phase II trials for advanced melanoma and cutaneous T-cell lymphoma as well as a phase I/II trial for refractory and relapsed B-chronic lymphocytic leukaemia.

CHS-828 (Figure 1), a pyridyl cyanoguanidine, is a small molecule inhibitor displaying cytotoxicity in a broad panel of cell lines [23]. We previously identified CHS-828 as an inhibitor of NAD synthesis [24]. We found CHS-828 to function similarly to APO866 in a number of assays although the two compounds are chemically distinct. Therefore, we suggested CHS-828 as an inhibitor of NAMPT. Furthermore, we compared a cell line, NYH/CHS, with acquired, specific resistance towards CHS-828 with its wild type counterpart without identifying the molecular basis for the resistance observed towards CHS-828 and APO866. CHS-828 has completed several phase I trials in oncology [25,26] and a prodrug EB1627/GMX1777 is currently also in phase I trials [27].

Here, we present a novel, potent analogue of CHS-828, namely TP201565 (Figure 1). This compound was found as part of a screen for NAMPT inhibitors and has shown activity in xenograft models. We developed a number of cell lines resistant to APO866 and TP201565. In most (4/5) of these we find mutations in the NAMPT gene that confer resistance when transfected into sensitive cells. The resistant cell lines show tumourigenicity in xenograft mouse models and *in vivo *resistance. Furthermore, through computer modelling and *in vitro *biochemistry we find that APO866, CHS-828 and TP201565 share a binding site in the active site of NAMPT, thus conclusively identifying CHS-828 and TP201565 as competitive inhibitors of NAMPT.

## Methods

### Drugs and chemicals

CHS-828 was synthesized as previously described [28]. APO866 and TP201565 and its linkable analogue were obtained from TopoTarget A/S (Copenhagen, Denmark). All other chemicals used in this paper were obtained from Sigma (St. Louis, MO, USA).

### Cell lines

NYH and NYH/CHS have been described previously [24]. HCT-116, PC-3 and HEK293T were obtained from ATCC. To obtain resistance towards NAMPT inhibitors, the cell lines were cultured in a medium containing the drug in gradually increasing doses. This process was continued over an extended period of time until a level of stable, high-grade resistance was reached.

### Clonogenic assay

Cells were incubated with the drug at different concentrations, in triplicates with a 3-week incubation period. Afterwards, the colonies were counted as described previously [29]. The colonies were counted in triplicate.

### Western blotting

Western blotting of NAMPT and GAPDH was performed as described before [24]. The anti-rabbit PBEF antibody (Bethyl Laboratories, Montgomery, TX, USA) was used for the detection of NAMPT at a dilution of 1:500. Densitometry measurements were done using Quantity One software (Bio-Rad Laboratories, Hercules, CA, USA) and results were averaged from 2-3 separate blots.

### Cloning of NAMPT

Total RNA was purified from NYH/CHS, NYH/APO866, HCT-116/APO866, HCT-116/TP201565 and PC-3/TP201565 using Trizol reagent (Invitrogen, Carlsbad, CA, USA). mRNA was converted to cDNA using TaqMan^® ^Reverse Transcription Kit (Applied Biosystem, Foster City, CA, USA) which was used as template for full length PCR of NAMPT by RED Extract-N-Amp™PCR ReadyMix™(Sigma). PCR products of all cell lines were sequenced twice and compared to NAMPT (GenBank accession # U02020). NAMPT flanked by BamHI/XhoI restriction sites was cloned into the TOPO TA cloning vector pCR 4-TOPO^® ^(Invitrogen) and clones containing identified mutations were selected. The mutated sequences were enzymatically cut with ScaI and BsaI (New England Biolabs, Ipswich, MA, USA) and ligated into wild type NAMPT in a pET28a(+) expression plasmid backbone (a kind gift from Dr. Shin-Ichiro Imai from Washington University School of Medicine, St. Louis USA) using T4 DNA ligase (New England Biolabs). Rosetta™2 (DE3) competent cells (Novagen, Merck, Darmstadt, Germany) were transformed with the resulting vectors. Sequencing was used to confirm correct insertion of the mutations.

### Transfection

NAMPTwt, NAMPTH191R, NAMPTD93del and NAMPTQ388R/K342R with BamHI/XhoI restriction sites were ligated into the mammalian expression vector TRex pcDNA-4/TO/Myc.His (Invitrogen). 100 μl sterile water containing 20 μg/ml vector was mixed with 4 μl FuGENE HD transfection agent (Roche, Basel, Switzerland). After 20 min the mix was added to 2 × 10^5 ^HEK-293T cells plated in 2 ml DMEM medium (Invitrogen) in 6-well plates 24 hours before transfection. After 24 hours, the cells were replated for experiments or for establishment of stable cell lines (HEK/CTR and HEK/NAMPTwt) in medium containing 300 ng/ml zeocin (Invitrogen). Cell proliferation was examined using CellTiterGlo (Promega, Madison, WI, USA) as described previously [24] using 72 hours incubation.

### Recombinant protein expression and determination of enzymatic activity of NAMPT

The proteins were expressed as previously described [12] using 5 hour induction at 37°C with 1.5 mM IPTG (Sigma) and lysis was done in 20 mM Tris-HCl pH 8.0, 300 mM NaCl, 0.1% Triton X-100 and protease inhibitor cocktail (Sigma) using sonication. His-Tagged NAMPT proteins were purified using nickel-nitrilotriacetic acid resin (Qiagen, Hilden, Germany) and imidazole elution. Purity and yield was evaluated by SDS gel electrophoresis and A280 measurements. Purified His-Tagged NAMPT proteins (WT, H191R and D93del) were incubated in reaction buffer (50 mM Tris-HCl [pH 8.5], 100 mM NaCl, 0.25 mM nicotinamide, 10 mM MgSO_4_, 1.5% ethanol, 0.5 mM phosphoribosyl pyrophosphate, 2.0 mM ATP) for 55 min at 37°C. The reaction was then coupled with NMNAT1 (cloned and expressed similarly to NAMPT - as above) and alcohol dehydrogenase (Sigma) 10 μg/ml each and incubated for further 5 minutes at 37°C. NMN production by NAMPT was evaluated indirectly by measuring the fluorescence (340 nm excitation, 465 nm emission). Fluorescence created by NADH was converted to NMN amounts using a standard curve with known amounts of NMN. k_cat _values were averaged from calculations of k_cat _for 500, 1000 and 2500 ng NAMPT enzyme. IC_50 _values for APO866 were obtained using the same assay setup as described above. For each enzymatic reaction 2000 ng purified NAMPT was used. The concentrations of inhibitor used to generate the inhibition curves were in the range from 0 nM - 0.5 μM for the wild type NAMPT and 0 nM - 100 μM for H191R.

### Xenograft studies

All mouse experiments were performed in accordance with national and EU legislation, and a permit to perform the specific type of experiments was obtained from the Experimental Animal Inspectorate, Danish Ministry of Justice. 10^7 ^cells of the paternal or resistant cell line of each of the four cell lines were injected s.c. in a mixture of PBS/matrigel in the right and left flank of female NMRI athymic mice (Taconic, Ry; Denmark), respectively. The mice were observed daily and tumour volumes were calculated from caliper measurements to estimate the radius and using the formula for a sphere. Tumours were allowed to reach a maximum volume of 1000 mm^3^, where after the mouse was sacrificed. Tissue samples of the xenograft tumours were excised and placed in phosphate buffered formaldehyde (4%, pH 7) for at least a week, and were then embedded in paraffin for sectioning and H&E staining. In xenograft studies with either the wild type or APO866-resistant variant of the HCT-116 tumour cell line mice were treated with a standard two daily i.p. injections of 15-20 mg/kg APO866 in PBS with 1% hydroxypropyl-β-cyclodextrin/12% propylene glycol on day 0-13, starting when tumour volumes were around 100 mm^3^, as previously described [30].

### Statistical analyses

Statistical analysis and the graphical presentation were performed using the software GraphPad Prism^® ^v. 4.0 (GraphPad Software, Inc., San Diego, CA, USA). Tumour doubling times were calculated using the line-fitting function for exponential growth in Graph Pad Prism, and were compared using Student's *t*-test. The outcome of xenografts in the treatment experiment was quantified as the number of days until the tumour of each individual mouse reached a size of 800 mm^3^, expressed as survival days. The survival in each treatment group was compared using log-rank analysis. The level of significance was set to a p value of 0.05.

### Measurement of NAMPT activity in lysates

Activity of NAMPT was examined in PC-3 and PC-3/TP201565 as well as HEK293T cells and HEK/NAMPTwt. NAMPT activity was measured by the conversion of ^14^C-labelled nicotinamide to ^14^C-NMN using a method previously described [31,32] using 50 μg total protein from cell lysate. Briefly, lysate was incubated with ATP, 5-phosphoribosyl-1-pyrophosphate and ^14^C-nicotinamide in reaction buffer. The mix was transferred to glass fibre filter and washed in acetone to remove excess ^14^C-nicotinamide. Counts per minute (cpm) determined by a liquid scintillation counter.

### Computer modelling of NAMPT

The molecular modelling was based on the published X-ray structure of NAMPT in complex with the inhibitor APO866 (2.10 Å resolution, PDB id: 2GVJ) [14]. The structure was imported into Maestro v. 9.0 release 111 and prepared for docking in the following way using the protein preparation wizard (Schrodinger Suite 2009, Schrodinger LLC, Oregon, USA). First, bond orders were assigned and hydrogen atoms were added. The four selenocysteines (Cys368 and Cys372 in both monomers) which had been incorporated to facilitate X-ray crystallographic analysis were mutated back to cysteine. Water molecules further than 5Å away from the binding site were removed, and finally the hydrogen bonding network was optimized. The Glide docking module was used for the docking of the ligands. Initially, the receptor grid was generated (no vdW scaling of the receptor atoms was applied). The enclosing box for the docking of TP201565 and CHS-828 was based on the position of APO866 in the X-ray crystal structure. The hydroxyl groups in the receptor were allowed to rotate. For studies on the H191R mutation, APO866 was removed in the 2GVJ X-ray structure which had already been prepared for molecular modelling. The Prime module allowed for introduction of the mutation, which was followed by sampling of Arg191 dihedral angles to obtain the most favorable orientation of the new side chain.

### Precipitation assays with sepharose linked analogue of TP201565

10 mg of the linkable analogue of TP201565 were coupled to NHS activated sepharose (GE-Healthcare) following the manufacturer's instructions using Na_2_CO_3_-containing reaction buffer and ethanolamine for blocking. HCT-116 cells were lysed in MMENGM buffer (25 mM MOPS pH[7,4], 2 mM EDTA, 2 mM sodium orthovanadate, 1 mM NaN_3_, 10% glycerol and 0,1% NP-40) with (1:200) protease inhibitor cocktail (Sigma) by sonication. Binding of NAMPT was conducted by mixing 30 μg of cellular protein with 20 μl coupled TP202648 beads and incubation for 60 min at 5°C with end over end rotation. After incubation, the beads were spun down and the supernatant removed followed by 3× washes with PBS (Invitrogen). Samples were boiled for 5 min at 95°C and run on a SDS gel followed by evaluation of the precipitated NAMPT protein by western blotting. For competitive studies, lysates were pre-incubated with APO866 (250 μM-2.5 nM) or TP201565 (250 μM-2.5 nM) for 15 min at 5°C before adding inhibitor linked beads and incubating for 60 min at 5°C.

## Results

### Development of resistant cell lines and identification of NAMPT mutations

We previously used the cell line NYH/CHS with resistance towards CHS-828 to determine the mechanism of action of CHS-828 as depletion of NAD, likely through inhibition of NAMPT [24]. From sensitive cell lines we developed a number of resistant cell lines to the NAMPT inhibitor APO866: NYH/APO866 and HCT-116/APO866, and to the CHS-828 analogue TP201565 (figure 1): HCT-116/TP201565 and PC-3/TP201565. Furthermore, we attempted to induce resistance in several other combinations of compounds and cell lines including NYH and HepG2 (hepatoma) cells with TP201565 and RPMI-8226 (myeloma) cells with APO866. No high-grade, stable resistance developed during 4 months of culture with drug (data not shown). Further, we have previously suggested that the resistance of NYH/CHS could be caused by a mutation in NAMPT [24]. Thus, we sequenced the NAMPT gene in the resistant cell lines and their parental lines. We found acquired mutations in NYH/CHS, NYH/APO866, HCT-116/APO866 and HCT-116/TP201565 but not in PC-3/TP201565 (Table 1). We also found two mutations that were present in both the HCT-116 parental cell line and in the resistant derivates. One, 903G->A, was silent (not shown) and previously identified as a SNP (rs11553095, dbSNP build 130) whereas the other, 1025A->G, resulted in a change in protein sequence of K342R. Interestingly, the mutations H191R and D93del occurred in both resistant cell lines of HCT-116 and NYH, respectively. We correlated the positions of the mutations in the protein sequence with their positions in a NAMPT crystal structure published previously [14]. We found that the acquired mutations were either located in the binding site of APO866 and nicotinamide or at the dimer interface of NAMPT whereas K342R was located distantly from either. All the mutations occurred in only one allele. In HCT-116/APO866 and HCT-116/TP201565, H191R and K342R were located on separate alleles whereas in the latter cell line, Q388R and K342R were located together on the same allele.

**Table 1:** Acquired and occurring mutations identified in NAMPT inhibitor resistant cell lines and parental line HCT-116.

	Mutation	Change in protein sequence	Position
***HCT-116***	1025A>G	K342R	b-sheet 11

***HCT-116/TP201565***	1025A>G	K342R	b-sheet 11
	1163A>G	Q388R	Dimer interface
	572A>G	H191R	APO866 binding site

***HCT-116/APO866***	1025A>G	K342R	b-sheet 11
	572A>G	H191R	APO866 binding site

***NYH/CHS***	278-280del	D93del	Dimer interface

***NYH/APO866***	278-280del	D93del	Dimer interface

The structural position of the mutations has been determined from a published crystal structure of NAMPT [14]. All mutations are located in one allele only. In HCT-116/TP201565 the mutations K342R and Q388R are located on the same allele.

We studied the LD_50 _values of the sensitive and resistant cell lines in a clonogenic survival assay. The parental cell lines were all sensitive with LD_50 _values of 1.5-11 nM for APO866 (Table 2). TP201565 was a more potent compound in this clonogenic assay compared to APO866 and CHS-828 (10-100 fold). Generally, the cell lines displayed a high degree of cross-resistance (Table 3). NYH/CHS and NYH/APO866 displayed similar resistance to the compounds (data not shown). Further, we found that the resistance observed for each cell line was retained after at least 15 weeks of culture without drug (data not shown). Interestingly, we observed LD50 values of 5-10 μM for CHS-828 in HCT-116/APO866, HCT-116/TP201565 and PC-3/TP201565 (Table 3) that were lower than for APO866. Otherwise, this compound shows similar or higher potency.

**Table 2:** Sensitivity of cell lines as measured in a clonogenic survival assay.

	APO866	CHS-828	TP201565
***NYH***	1.5 ± 0.5	1.7 ± 1.2	0.02 ± 0.01

***HCT-116***	10.9 ± 6.1	12.2 ± 7.4	0.4 ± 0.3

***PC-3***	3.8 ± 3.1	8.8 ± 8.4	0.3 ± 0.3

LD_50 _values (measured in nM) of the parental cell lines NYH, HCT-116 and PC-3 when treated with APO866, CHS-828 or TP201565. LD_50 _values are represented with standard deviations.

**Table 3:** Fold increase of LD_50_ in resistant cell lines for treatment with NAMPT inhibitors.

	APO866	CHS-828	TP201565
***NYH/CHS***	18.3	168	33.5

***HCT-116/APO866***	946	414	1,723

***HCT-116/TP201565***	2,364	774	10,103

***PC-3/TP201565***	8,324	624	19,675

The level of resistance of the resistant cell lines, NYH/CHS, HCT-116/APO866, HCT-116/TP201565 and PC-3/TP201565 is shown for treatment APO866, CHS-828 and TP201565. Values represent the ratio of LD_50 _of the resistant cell line:LD_50 _of parental cell line as measured by clonogenic survival assay.

**Figure 2:**
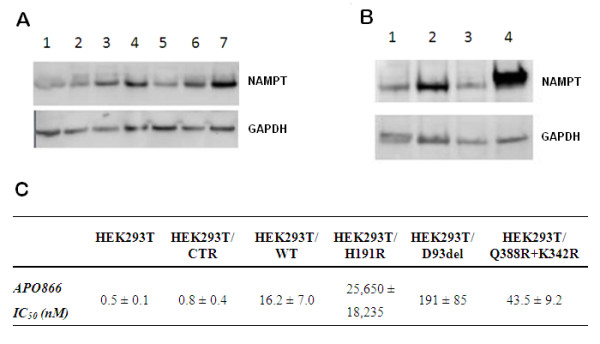
**NAMPT expression in tumor cell lines and transfected HEK293T cells**. **A: **Protein expression of NAMPT in parental and resistant cell lines. **1-7: **NYH, NYH/CHS, HCT-116, HCT-116/APO866, HCT-116/TP201565, PC-3 and PC-3/TP201565. **B: **Comparison of protein expression of NAMPT in **1 **PC-3, **2 **PC-3/TP201565, **3 **HEK293T cells transfected with control vector and **4 **HEK293T cells transfected with an expression vector containing the NAMPT gene. The expression is representative for transfections with wild type and mutant NAMPT. GAPDH is used as loading control. **C: **IC_50 _values of APO866 in HEK293T cells, untransfected and transfected with control vector, wild type NAMPT and NAMPT containing H191R, D93del or Q388R/K342R. Values are shown in nM ± SD.

We also studied the protein expression of NAMPT in the parental and resistant cell lines. We found a slight up-regulation around 20% of NAMPT in HCT-116/APO866 compared to HCT-116 (figure 2A). Further, we observed higher levels of NAMPT in PC-3 cells compared to other parental cell lines and a marked up-regulation in PC-3/TP201565 compared to PC-3 (around 80%).

### Over-expression of NAMPT variants induce APO866 resistance in sensitive HEK293T cells

To examine whether the acquired mutations are primarily responsible for the resistance observed in the resistant cell lines we over-expressed wild type NAMPT (NAMPTwt) and NAMPT containing either the H191R, D93del or Q388R/K342R mutations in HEK293T, a sensitive cell line, and measured the resistance induced by the NAMPT over-expression. We created a robust over-expression of NAMPT (Figure 2B) and we found that NAMPTwt induced an increase of resistance towards APO866 by 20 fold (p < 0.005). A further relative increase in resistance was induced by all mutant NAMPTs with the order of induced resistance being H191R>D93del>Q388R/K342R (Figure 2C). Over-expression of NAMPT containing the Q388R/K342R, H191R or D93del mutation yielded significantly greater resistance compared with wild type NAMPT over-expression (p = 0.007, p = 0.01 and p = 0.002, respectively). The H191R and D93del mutations resulted in an induced resistance in HEK293T cells towards APO866 similar to or above that of the corresponding resistant cell line. No significant difference was observed between untransfected HEK293T cells and HEK293T/CTR (empty vector).

### APO866 binding and enzyme activity is affected by NAMPT mutations in vitro

**Figure 3:**
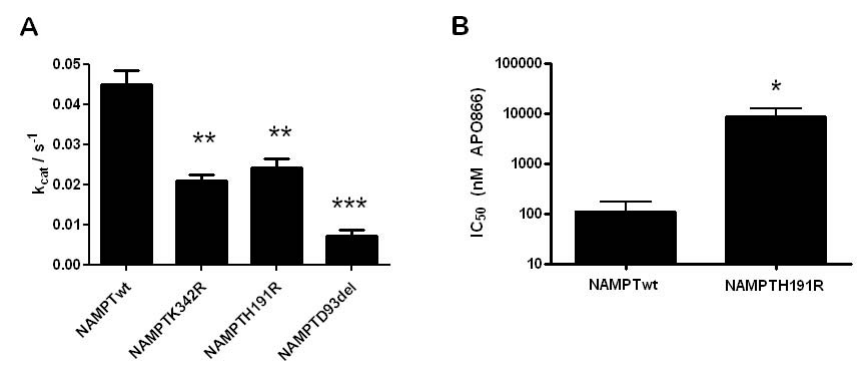
***In vitro *NAMPT enzyme assays**. **A: **k_cat _values for wild type NAMPT (NAMPTwt) and NAMPT with K342R, H191R and D93del mutations respectively. Statistical significance compared with NAMPTwt: **: <0.0005, ***: <0.0001. **B: **IC_50 _values of APO866 for in vitro inhibition of NAMPTwt and NAMPTH191R. The Y-axis is logarithmic. Statistical significance compared with NAMPTwt: *: <0,05. Error bars indicate SEM.

To study further the H191R and D93del mutations we purified recombinant NAMPTwt and NAMPT with either mutation. Using a colorimetric, coupled assay we determined the k_cat _for NAMPTwt (measured per active site), NAMPTH191R and NAMPTD93del. We found that the activities of NAMPTH191R and NAMPTK342R were reduced by ~50% by the mutations when compared to wild type whereas NAMPTD93del displayed 20% of the wild type activity (Figure 3A). We examined the inhibitory effect of APO866 in NAMPTwt and NAMPTH191R. The IC_50 _value for inhibition of NAMPTwt was 110 nM while we found an IC_50 _value for NAMPTH191R of 8585 nM (Figure 3B). Further, we found that APO866, CHS-828 and TP201565 did not inhibit NMNAT (data not shown).

### NAMPT mutations do not inhibit in vivo xenograft growth and maintain resistance towards APO866

**Figure 4:**
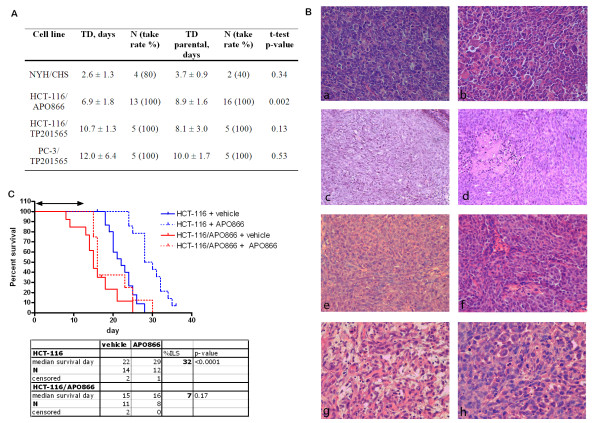
**Subcutaneous xenograft experiments of parental and resistant cell lines**. **A: **Tumour doubling times of NAMPT resistant cell lines compared to tumour doubling times (TD) of parental cell lines (shown with SD). p-values are determined by *t*-tests. **B: **H&E stained sections of xenograft tumours at 200× magnification. **a **NYH and **b **NYH/CHS xenografts contained cells of various sizes and showed many mitoses. Tumours of **c+e **HCT-116, **d **HCT-116/APO866 and **f **HCT-116/TP201565 contain many fine connective tissue strands between tumour cells. Also, necrotic foci and densely growing epithelial cells are observed. Morphology of **g **PC-3 and **h **PC-3/TP201565 varied between areas of rounded cells and more spindle-shaped tumour cells. Many pyknotic (apoptotic) cell nuclei were also found. **C: **Cumulative survival of mice with HCT-116 and HCT-116/APO866 xenografts showing the time used by each mouse to grow its tumour to a size of 800 mm^3^. The mice were treated with 15-20 mg/kg APO866 or vehicle twice daily for 14 days (0-13, double arrow), where day 0 indicates the day tumours had a size of approx. 100 mm^3^. The result of log-rank analysis comparing APO866 treated mice with control mice in the respective xenografts (HCT-116: blue, HCT-116/APO866: red) is shown on the graph.

We studied whether the resistance induced *in vitro *would have an effect on tumour viability *in vivo*. Xenograft models were performed for parental cell lines NYH, HCT-116 and PC-3 as well as their resistant derivates, and tumour formation rates were not affected in the resistant cell lines compared with parental cell lines. NYH tumours had a very fast doubling time in xenografts (3.7 days) followed by HCT-116 and PC-3 (8.9 and 10 days respectively). We observed no significant change in tumour doubling times for the resistant cell lines when compared to their wild type counterparts (Figure 4A). However, HCT-116/APO866 had a shorter tumour doubling time, 6.9 ± 1.8 days, compared with HCT-116 (8.9 ± 1.6 days) (Figure 4A). We examined tumour tissues histologically and found no obvious morphological differences between untreated sensitive xenografts and their resistant counterparts (Figure 4B). Notably, although xenograft tumours from NAMPT inhibitor-resistant derivatives of HCT-116 were found to contain many necrotic areas, similar results were observed for parental HCT-116 cells. Finally, we treated HCT-116 and HCT-116/APO866 xenografts with 20 mg/kg APO866 daily for 14 days. We observed that APO866 increased the lifespan significantly in HCT-116 xenografts, increasing the median time for a tumour to reach the size of 800 mm^3 ^with 32% from 22 to 29 days, whereas treating the HCT-116/APO866 xenografts with APO866 resulted in no effect on the survival (Figure 4C), confirming *in vivo *resistance. There were no differences in body weight between the groups (data not shown).

### PC-3/TP201565 and HEK293T/WT display high NAMPT activity compared to PC-3 and HEK293T

**Figure 5:**
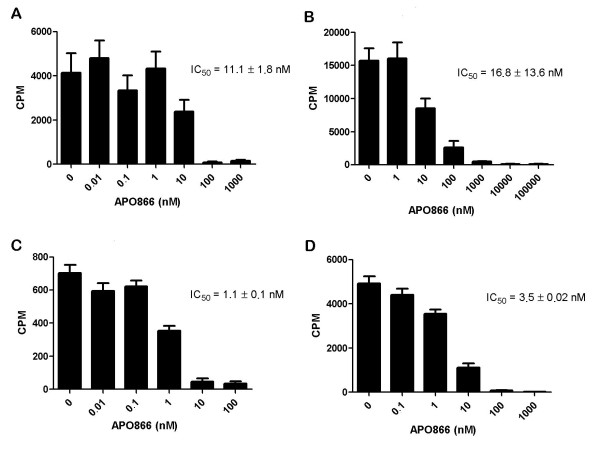
**Total *in vitro *activity of NAMPT in cell lysates**. NAMPT activity as measured by conversion of C14-labelled nicotinamide to NMN by 50 μg of total protein from lysate untreated or with increasing concentrations of APO866. Absolute counts per minute (CPM) are presented. The IC_50 _values have been averaged from at least 3 individual experiments. Cell lines measured are **A **PC-3, **B **PC-3/TP201565, **C **HEK293T and **D **HEK293T/WT. Basal activity of PC-3/TP201565 is 4.0 fold higher than PC-3 and basal activity of HEK293T/WT is 7.4 fold higher than HEK293T. Error bars indicate SEM; IC_50 _values are represented with standard deviations.

Since PC-3/TP201565 cells contained no mutations in NAMPT we examined whether the increased protein expression could be responsible for the NAMPT inhibitor resistance observed in the cell line. We studied the total *in vitro *activity of NAMPT from lysates of PC-3 and PC-3/TP201565 untreated and in response to inhibition with APO866. We found that the basal total activity of NAMPT was four fold higher in the resistant line compared to PC-3 in accordance with the higher expression of NAMPT (Figure 5A+B). However, the IC_50 _value for *in vitro *lysate treatment with APO866 was not significantly increased. Similarly, we observed a strong increase in total NAMPT activity when over-expressing NAMPT in HEK293T cells compared with wild type HEK293T cells (Figure 5C+D). Likewise, the IC_50 _was not increased by over-expression although the absolute NAMPT activity remained elevated at concentrations above the IC_50 _compared with untreated wild type HEK293T cells. Respective protein expression levels of NAMPT for the cell lines are shown in Figure 2B for comparison. Finally, we detected no up-regulation of MDR 1 and 2 pumps at the mRNA level and we found that PC-3/TP201565 cells show no cross-resistance towards the histone deacetylase inhibitor belinostat, the proteasome inhibitor MG-132, the DNA methyltransferase inhibitor 5-azacytidine, the topoisomerase I inhibitor camptothecin, the toposiomerase II inhibitors and ABC substrates doxorubicin and mitoxantrone, retinoic acid, the IKK inhibitor BMS-345541 or the protein kinase inhibitor staurosporine (data not shown). The drugs were chosen to cover a broad spectrum of mechanisms of action (including drugs sensitive to MDR mechanisms) to give an indication of the molecular basis of resistance in PC-3/TP201565 cells.

### Docking studies confirm that APO866, CHS-828 and TP201565 share a common binding site

To study the relationship between the active site of NAMPT and APO866, CHS-828 and TP201565 as well as the H191R mutation, we used computer modelling based on a published x-ray structure of NAMPT [14]. The x-ray crystal structure contains several water molecules located either within or close to the binding site, hence we decided to carry out two separate docking runs; one in which water molecules within 5Å of the APO866 binding site were included in the calculations, and another without water molecules. Traditionally, docking has been carried out using water-free enzyme active-site, but recently with increased accuracy of crystal structures and increased predictivity of computational chemistry the importance of water molecules has been realized [33]. If two ligands have very similar binding modes one might therefore investigate whether the crystal waters observed experimentally are also suitably positioned for the ligand where no crystal structure is available.

**Figure 6:**
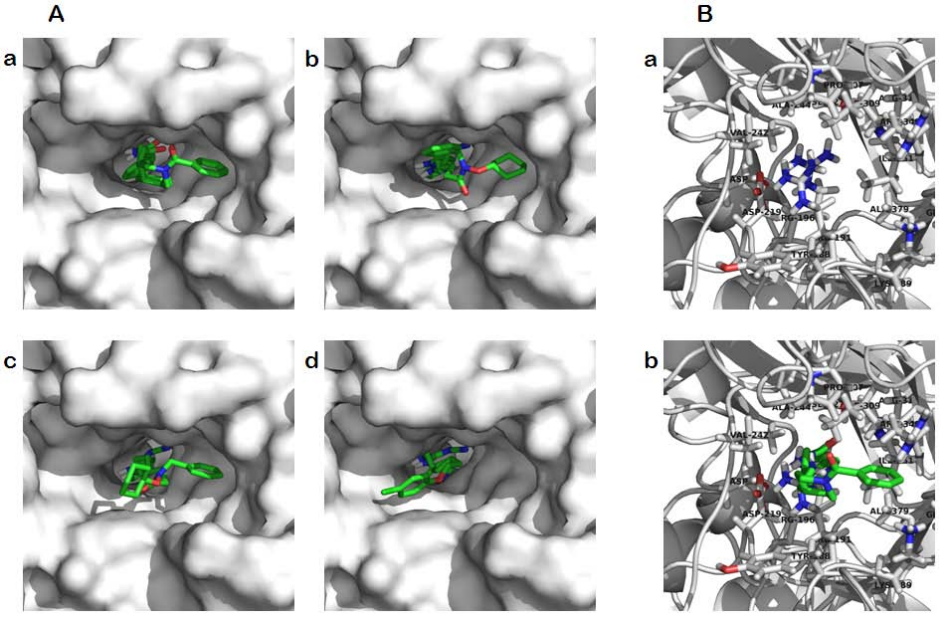
**Computer modelling of NAMPT**. **A **Binding of inhibitors in wild type NAMPT. **a **Binding of APO866 from x-ray structure and computer modelling (XP glidescore -8.92) in the NAMPT active site superimposed. **b **The best docking of TP201565 in NAMPT with structural water present in the active site XP (glidescores -8.29). **c **Alternative docking of TP201565 in the active site of NAMPT with switched positions of the phenyl and cyclo-hexane groups (XP glidescore -7.53). **d **Docking of CHS-828 in NAMPT without structural water present (XP glidescore -7.23). **B **Modelling of H191R. **a **The Arg191 of NAMPTH191R protrudes into the active site of NAMPT. **b **APO866 docking in the active site of NAMPTH191R.

When the water molecules were present in the binding site, the highest-ranking docking conformation of APO866 was in good agreement with the reported X-ray structure (RMS 0.2003Å for non-hydrogen atoms, XP glidescore -8.92, Figure 6Aa). Surprisingly, also TP201565 was able to dock into the solvated active site with the pyridine moiety in the same π-stacking binding pocket between Tyr18' and Phe193. The double-headed TP201565 molecule could be stabilized by displaying either the phenyl or the cyclo-hexane in the opening of the narrow pore where APO866 has a phenyl group (figure 6Ab+c, XP glidescores -8.29 and -7.53, respectively). For CHS828 we did not obtain any docked conformations with good XP glidescore (lower than -6), indicating that the water-filled active site suitable for APO866 is not compatible with this compound.

**Figure 7:**
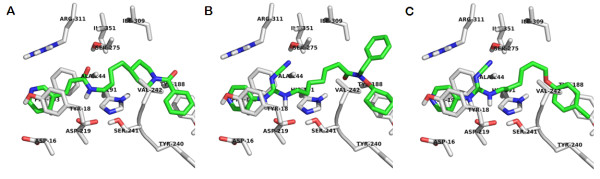
**Computer modelling of NAMPT without structural water present**. **A **Top-view representation of APO866 binding. **B **Top-view representation of TP201565. **C **Top-view representation of CHS-828.

Then, we turned our attention to the docking experiments carried out using the empty binding site without water molecules. The best scoring conformation of APO866 now had the phenyl group pointing towards the other pocket (XP glidescore -8.18, shown as top view, Figure 7A), but a conformation similar to the crystal structure was recovered as the fifth best conformation (XP glidescore -6.98, data not shown). TP201565 was able to achieve a good conformation (XP glidescore -7.78, top-view, Figure 7B). With the water molecules removed from the binding site CHS-828 was also able to obtain good interactions (XP glidescore -7.23, top-view, Figure 7C). All three compounds had the pyridine substituent sandwiched between Tyr18' and Phe193. The change from the alkene group in APO866 to the guanidinium group in TP201565 and CHS828 gave rise to a H-bond between the NH-group proximal to the pyridine and Asp219. While this caused these ligands to move closer to Asp219 the long cyano-group still allowed the formation of a H-bond to Ser275. The overall position of CHS-828 in NAMPT without structural water present in the binding site is shown in Figure 7D.

Further, we studied the effect of the H191R mutation on binding APO866 in the active site. We found that Arg-191 when modeled into NAMPT protruded into the active site and sterically completely blocked binding of APO866 (Figure 6B) as well as CHS-828 and TP201565 (data not shown).

### TP201565 is a competitive inhibitor of NAMPT in vitro

**Figure 8:**
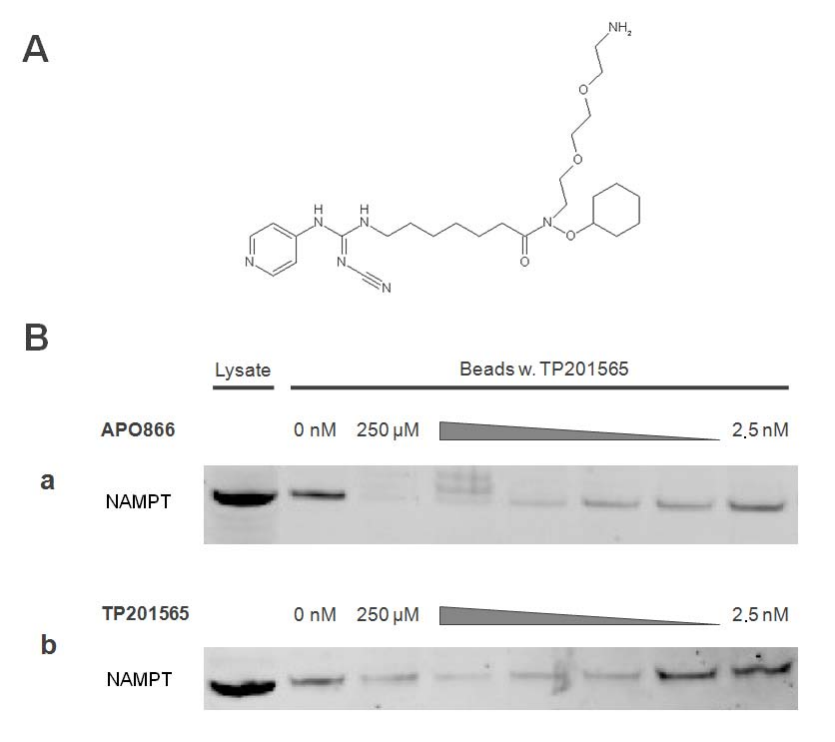
**Precipitation of NAMPT by sepharose linked TP201565**. **A: **Chemical structure of linkable TP201565 analogue **B: **This figure shows western blots of NAMPT from 20 μg protein from HCT-116 lysate as well as NAMPT precipitated from HCT-116 lysate containing 20 μg total protein by TP201565 linked covalently to sepharose beads. The binding of NAMPT to beads is performed either with no inhibitor or in competition with **a **APO866 or **b **TP201565 at concentrations between 250 μM and 2.5 nM (in 10-fold steps).

To confirm the notion gained from the computer modelling results that CHS-828 and its analogues are competitive inhibitors of NAMPT we used a modified analogue of TP201565 that contained a long carbon linker with a reactive amine (Figure 8A). The analogue retained cytotoxic potency within 10 fold of unmodified TP201565 (data not shown). The analogue was coupled covalently to sepharose beads and used for protein precipitation from cellular lysate of HCT-116 cells. We found that linked TP201565 could bind and precipitate NAMPT from lysate for analysis by western blot (Figure 8B). Pre-incubation of the lysate with APO866 blocked the precipitation of NAMPT suggesting that the binding sites of APO866 and TP201565 are overlapping. Since blocking NAMPT precipitation required relatively high concentrations of APO866 we examined the blocking effect of unmodified TP201565. We found that unmodifiedTP201565 was required in roughly the same concentrations for an effective abrogation of NAMPT precipitation (Figure 8B) indicating similar affinities for the binding site.

## Discussion

Although CHS-828 has been established as an inhibitor of NAD synthesis with a mechanism of action similar to APO866, it had not been definitively identified as an inhibitor of NAMPT. Furthermore, previous attempts to identify resistance mechanisms for CHS-828 have yielded no definite results [24,34] and resistance to APO866 has not been studied. In this study we introduce a novel potent analogue of CHS-828, TP201565, that shows >10 fold increased activity in sensitive cancer cell lines compared to APO866 and CHS-828. Using computer modelling, we find that CHS-828 and TP201565 most likely function as inhibitors of NAMPT by binding to the same site as APO866 and nicotinamide. This is further supported by the fact that a sepharose linked TP201565 analogue can precipitate NAMPT from cellular lysate and that this binding can be abrogated by co-incubation with either APO866 or TP201565. We also show how mutations in NAMPT can block the binding of APO866, CHS-828 and TP201565. Together, these data conclusively determine the pyridyl cyanoguanidines, CHS-828 and TP201565, as competitive inhibitors of NAMPT. This is in agreement with very recently reported data, which through the use of metabolic screens, *in vitro *NAMPT assays and computer modelling reach a similar conclusion for CHS-828 [35].

We developed four novel cell lines with acquired drug resistance towards NAMPT inhibitors and including NYH/CHS cells we observed mutations in all except PC-3/TP201565. Interestingly, the H191R mutation was detected in two different resistant cell lines derived from HCT-116. Furthermore, the distinct deletion D93del was located in both NYH/CHS and NYH/APO866. This suggests that these mutations exist in a small subpopulation of the parental cell line and that in these cases resistance has been obtained from selection rather than *de novo *mutations. The fact that we never obtained resistance from treating NYH cells with TP201565 despite prolonged growth with drug may be due to the fact that cells containing the NAMPTD93del were not present in the original flask used for inducing resistance to TP201565. We found that the observed mutations in NAMPT could induce resistance similar to or above that seen in the original resistant cell line which strongly suggests the mutations as the primary mechanistic cause of resistance. Furthermore, we demonstrated that whereas H191R abrogated NAMPT inhibitor binding in computer modelling it only reduced the enzymatic NAMPT activity *in vitro *by 50% - similar to what we found for the K342R mutation. The change in k_cat _may be due to altered substrate affinity resulting in an increased K_M _value. Also, we showed that the H191R mutation strongly increased the IC_50 _value for APO866 inhibition *in vitro*. The relatively high IC_50 _value of NAMPTwt obtained *in vitro *correlated with the previous results of others [35]. D93del on the other hand reduced k_cat _*in vitro*. Although this may reduce overall NAMPT activity compared to wild type NYH cells, it is likely that enough NAD is produced to keep up with the cellular demands and thus no growth disadvantage is inferred. NYH/CHS cells have previously been shown to retain the D93del mutation and full resistance after 30 passages without drug present in the media [24] (unpublished results, Olesen, UH). Similarly we have observed retained resistance for the remaining resistant cell lines after culture for more that 15 weeks without drug. We speculate that the induction of resistance by D93del and Q388R may be due to interference with or abrogation of dimerisation of NAMPT as both are located in the dimer interface. The NAMPT monomer may potentially retain lower enzymatic activity while not binding the known NAMPT inhibitors. Alternatively, the resistant cell lines may have adapted through reduced NAD consumption or up-regulation of other sources of NAD such as *de novo *synthesis.

Besides the data on the H191R mutation in figure 6B, the observed difference in kinetics and inhibitory constants between the different mutants cannot reliably be predicted on the basis of the computer docking study performed in this study. To achieve such correlations molecular dynamics calculations of the entire enzyme (including specific solvation) for tens of nanoseconds would be necessary. However, this is far beyond what is feasible with our current computational resources.

Notably, CHS-828 seems to show an upper level in LD_50 _values around 5-10 μM which is significantly lower than what is seen for APO866. We speculate that this may be due to a secondary mechanism of cytotoxicity occurring at above 1 μM. This has also previously been suggested [36,37]. Although this currently unknown mechanism may be less important in cellular and molecular assays, it may well be of interest clinically for CHS-828, specifically since serum concentrations of the drug have been reported to reach 11 μM in clinical trials [26]. However, it is unlikely that it is related to the previously proposed action of CHS-828 as an inhibitor of the I-κB kinase (IKK) complex [38]. CHS-828 showed no inhibition of IKKα, IKKβ or TBK1 enzymes *in vitro *(unpublished results, Olesen, UH). APO866 displayed a surprisingly specific induction of cell death in tumour cells ranging five orders of magnitude from close to 1 nM to more than 30 μM *in vitro *and a similar specificity was seen for TP201565. Finally, the specific nature required of mutations in NAMPT to confer resistance combined with the lack of resistance induced even at prolonged drug treatment in a number of combinations of cell lines and NAMPT inhibitors may indicate that resistance is not induced frequently unless a suitable mutation in NAMPT is already present in a population of tumour cells. However, we also found that the resistance is not easily reversible. Also, no significant increase in tumour doubling times occurred *in vivo *for the resistant cell lines. Rather, we find that HCT-116/APO866 xenografts displayed reduced tumour doubling times compared to HCT-116. As we did not observe a similar trend for PC-3/TP201565 it seems unlikely to be due to increased NAMPT levels. Rather, the induction of resistance may have resulted in the development of an unidentified growth advantage in HCT-116/APO866. Further, the *in vitro *resistance also conferred insensitivity to APO866 treatment in xenograft models shown for HCT-116/APO866.

The PC-3/TP201565 cell line is highly resistant towards NAMPT inhibitors while displaying up-regulation but no mutations of NAMPT. The up-regulation could be due to increased gene copy number. We found that the basal NAMPT activity in the resistant cell line and also in transfected NAMPT over-expressing HEK293T/WT was much higher than in wild type PC-3 and HEK293T cells. The fact that PC-3/TP201565 cells displayed higher total activity than HEK293T/WT despite the latter having higher expression of NAMPT agrees with previous findings that endogenous NAMPT has higher activity compared to recombinant NAMPT [39]. However, the IC_50 _was not significantly changed and although the absolute NAMPT activity remained high relative to wild type PC-3 and HEK293T at concentrations of APO866 up to 1-10 μM, it would not seem to fully explain the >10 μM LD_50 _value observed for APO866 in PC-3/TP201565. Further, the resistance appeared to be specific to NAMPT inhibitors as cross-resistance to other chemotherapeutics was not observed and it was not related over-expression of MDR1 and 2. Also, CHS-828 has displayed only low to moderate sensitivity to mechanisms of MDR based on over-expression of Pgp170 and MRP, increased levels of gluthation and tubulin-associated MDR [23,40]. Thus, we believe that PC-3/TP201565 cells are likely to possess a further, yet unknown, specific mechanism of resistance. Still, the over-expression explains part of the resistance and, as also seen for HEK293T/WT cells, high levels of NAMPT may be sufficient to induce resistance which would be significant in a clinical setting. This is similar to the situation with thymidylate synthase where high expression levels predict resistance to 5-fluorouracil and a poor treatment outcome [41,42].

The crystal structure of NAMPT in complex with APO866 has previously led to suggestions on improving the potency of APO866 based on its interaction with the NAMPT binding site [14,43]. So far, published APO866 analogues have shown less activity in cellular assays [44,45]. We found that the mutations in NAMPT identified in the resistant cell lines show a tendency to locate either in the binding site or dimer interface. These mutations may directly or indirectly adjust the shape of the binding site so that it is unsuitable for the relatively large NAMPT inhibitors while still accessible for nicotinamide and NMN. Also, others have recently published a single resistant cell line showing a point mutation, G217R, which is close to the active site of NAMPT and is responsible for high-grade resistance towards CHS-828 [14,35]. We hope that the identification of a new class of NAMPT inhibitors and the characterization of resistance inducing mutations in NAMPT may be useful in developing second-generation NAMPT inhibitors with higher potency and potentially less affected by acquired resistance.

## Conclusions

We have identified the cyanoguanidines CHS-828 and TP201565 as direct inhibitors of NAMPT. Further, we found that NAMPT mutations located around the enzyme active site or the dimer interface could be expected to be the most frequent cause of acquired resistance towards inhibitors of NAMPT. The location of the mutations could be applied as guide for development of next-generation NAMPT inhibitors with reduced risk of development of resistance.

## Abbreviations

(NAMPT): Nicotinamide phosphoribosyltransferase; (PARPs): poly-(ADP-ribose) polymerases; (NMN): nicotinamide mononucleotide; (NMNAT): NMN adenyltransferase; (NAMPTwt): wild type His-tagged NAMPT;

## Competing interests

TopoTarget A/S has funded parts of this study. UHO, JGP, MKC, AT, FB, PBJ, SJN and MS have recently been fully or partially employed by TopoTarget A/S and several authors also hold stock in TopoTarget A/S. TopoTarget A/S has licensed the rights to APO866 and owns the rights to TP201565 and has provided the drugs used for this study.

## Authors' contributions

UHO has been involved in the design and planning of the *in vitro *and *in vivo *experiments, carried out identification of mutations and cellular transfections and drafted the manuscript. JGP carried out recombinant NAMPT experiments and linker-assays. AG and WK designed and carried out the *in vitro *lysate activity studies. JY and SI generated the expression plasmid for wild type human NAMPT protein and contributed to the construction of plasmids for mutant proteins. MKC and FB synthesized the involved compounds. PF performed the *in silico *studies. AT carried out *in vivo *studies. SJN and MS were involved in the planning of the study and revised the manuscript and PBJ likewise revised the manuscript critically. All authors have read and approved the manuscript.

## Pre-publication history

The pre-publication history for this paper can be accessed here:

http://www.biomedcentral.com/1471-2407/10/677/prepub
